# Rearranging the Bird’s Nest Fungi: molecular review of internal clades in *Cyathus* (*Nidulariaceae*, *Basidiomycota*)

**DOI:** 10.1186/s43008-023-00111-y

**Published:** 2023-04-07

**Authors:** Rhudson Henrique Santos Ferreira da Cruz, Jefferson dos Santos Góis, Paulo Marinho, Iuri Goulart Baseia, Kentaro Hosaka

**Affiliations:** 1 Centro das Ciências Biológicas e da Saúde, Universidade Federal do Oeste da Bahia, Barreiras, Bahia Brazil; 2 Departamento de Botânica e Zoologia, Programa de Pós-Graduação em Sistemática e Evolução, Universidade Federal do Rio Grande do Norte, Natal, Rio Grande do Norte Brazil; 3 Departamento de Biologia Celular e Genética, Universidade Federal do Rio Grande do Norte, Natal, Rio Grande do Norte Brazil; 4 Department of Botany, National Museum of Nature and Science, 4-1-1 Amakubo, Tsukuba, Ibakari Japan

**Keywords:** Gasteromycetes, ITS-LSU analysis, Infrageneric classification

## Abstract

The genus *Cyathus* was established in 1768, but more in-depth taxonomic studies with the group only occurred after 1844. In the following years, changes in the infrageneric classification of *Cyathus* were proposed based mainly on morphology. With advances in phylogenetic studies, the morphological classifications were tested and a new subdivision into three groups was proposed in 2007. Based on the last two classifications, this work aims to expand and understand the internal phylogenetic relationships among the fungi of the genus *Cyathus* and examine how these relationships are reflected in the taxonomic classification, through molecular analyses covering most of the species in the group, based on materials obtained from type specimens deposited in major fungal collections worldwide, besides expanding sampling with tropical species. Molecular analyses followed the protocols available in the literature, including the design of specific primers for *Cyathus*. In the phylogenetic analysis, using Maximum Parsimony and Bayesian methods, sequences of ITS and LSU regions from 41 samples of 39 species of *Cyathus*, 26 were placed with some nomenclatural types. The monophyly of *Cyathus* was confirmed with maximum support in both tests, and the infrageneric groups of the most recent classification were unchanged, but the clade *striatum* showed segregation into four groups and three subgroups. The phylogenetic organization is supported morphological characters, and diagnoses are presented for each group, as well as a dichotomous key for the infrageneric separation.

## INTRODUCTION

*Cyathus* is a genus of gasteroid fungi characterized by their cone or inverted bell-shaped basidiomata, with the presence of lenticular structures attached to the inner wall of the basidiome, called peridioles (Brodie 1975). These structures resemble small eggs inside a bird’s nest, an analogy that has made the group known generically as bird’s nest fungi. According to Brodie (1975, 1984) and Lloyd (1906), most *Cyathus* species are cosmopolitan, but a few are endemic.

Structurally, the basidiomata of three of the five genera of bird’s nest fungi, namely *Cyathus*, *Crucibulum*, and *Nidula*, present with the shape of a cone or inverted bell. The other genera in the group, *Nidularia* and *Mycocalia*, have a globose or subglobose shape, being basically a cluster of peridioles covered by a thin peridium. In *Cyathus*, the peridium may or may not have longitudinal markings internally, externally or in both walls, called “plications” or “striae”, a characteristic that distinguishes it from *Crucibulum* and *Nidula*, which have a smooth peridium (Brodie 1975). Another difference between *Cyathus*, *Crucibulum,* and *Nidula* is found in the peridiole: during the splash-cup dispersion process, the peridiole of *Cyathus* adheres to vegetation exclusively through sticky hyphae at the end of its complex funicular cord, called hapteron. *Crucibulum* has a simple funicular cord with little adhesion, but the surface of the peridiole is also slightly adhesive, allowing the fixation to occur through both structures together. In the case of *Nidula*, the peridioles are small and without a funicular cord, but the structure itself presents high adhesiveness (Brodie 1975).

The first record of a probable *Cyathus* was made in 1601 by the botanist Carolus Clusius, who identified the peridiole sample only as an “anonymous and small fungus”. After years with few references about the group, the genus *Cyathia* was established by P. Browne in 1756, while the name *Cyathus* was proposed by Haller only in 1768 (White 1902) but is retained as it was sanctioned by Persoon (1801). After the nineteenth century, the group received a significant increase in knowledge, through the studies made by Tulasne and Tulasne (1844), who subdivided the genus into sections *Eucyathus* and *Olla*, based on the presence or absence of striae on the basidiome, respectively.

Lloyd (1906) proposed changes to this classification, separating the species into five subsections without names: two of *Eucyathus*, by the presence or absence of a tunica, and three of *Olla*, by the type of hyphal covering of the basidiomata (hirsute, woolly, or non-existent/glabrous). The latest morphological classification was proposed by Brodie (1975), who redistributed the species into seven groups according to the patterns of striae on the peridium, hyphal cover, presence or absence of tunica, and color of the basidiomata (Table 1).

**Table 1:** Morphological characteristics of the *Cyathus* groups proposed by Brodie (1975)

Group	Morphological characteristics
Group I—*olla*	Peridium expanded in the upper portion, not plicated, smooth-textured, with fine tomentum, usually shorter. External wall not contrasting with the inner wall. Basidiospores mostly ovoid, thin-walled, and with a thin tunica
Group II—*pallidus*	Peridium mostly light colored, not expanded abruptly in the mouth, not plicated. External wall woolly, with the long tomentum conspicuously directed downward. Smooth endoperidium, basidiospores mostly ovoid, thin-walled, and with a thin tunica
Group III—*triplex*	Peridium mostly dark colored, sometimes with an inconspicuous plication only in the endoperidium. Exoperidium with long hairs, sometimes aggregated into tufts. Smooth endoperidium, white or silver. Subhomogeneous cortex or distinctly double. Basidiospores ellipsoid, thick-walled, tunica thin or absent
Group IV—*gracilis*	Peridium not plicated, tomentum aggregated into tufts, basidiospores ellipsoid, thick-walled, and thin tunica
Group V—*stercoreus*	Peridium not plicated, numerous tomentum hair, irregular in length, woolly in appearance. Peridioles black or darker, tunic absent, basidiospores large and subglobose
Group VI—*poeppigii*	Exoperidium and endoperidium distinctly plicated, tomentum hirsute, strongly brownish. Peridioles dark to black, basidiospores large, globose, or ellipsoid. Tunica absent
Group VII—*striatus*	Endoperidium distinctly plicated. Exoperidium sometimes inconspicuous, with tomentum hirsute to woolly. Basidiospores mostly elliptical, tunica present

According to Brodie's morphological classification (Brodie 1975, 1984), the bird’s nest fungi are grouped in the order *Nidulariales* of the artificial group *Gasteromycetes*, due the presence of angiocarpic basidiomata and passive spore dispersal. Currently, the class *Gasteromycetes* is considered devoid of taxonomic significance because it is an artificial group that comprises fungi of distinct evolutionary lineages (Hibbet et al. 1997; Wilson et al. 2011). According to Hibbet et al. (1997) and Moncalvo et al. (2002), based on molecular data, fungi of the order *Nidulariales* were arranged within *Agaricales*, in an uncertain position, although included in *Agaricaceae* by Vellinga et al. (2011). Matheny et al. (2006) suggested the monophyly of the species of “bird’s nest fungi'', which further supports the *Nidulariaceae* as a monophyletic group within *Agaricales*, with tribe *Cystodermataceae*, family *Cystodermataceae*, as its sister group.

Phylogenetic studies focused on *Cyathus* have been carried out by Zhao et al. (2007), using the ribosomal DNA molecular markers ITS and LSU, with the aim to redefine the evolutionary patterns within the genus and to verify if the seven-group subdivision proposed by Brodie (1975), based on peridium plication and the tomentum patterns on the basidioma, is valid. The study used 115 samples with representatives of 22 species distributed among Brodie’s seven groups, including sequences of type specimens or species correctly identified by the authors, representing approximately one third of the 64 species validly published at the time, according to the online databases Mycobank (www.mycobank.org) and Index Fungorum (www.indexfungorum.org), and excluding synonymized and invalid records according to the International Code of Nomenclature of Algae, Fungi and Plants (Turland et al. 2018).

The results of Zhao et al. (2007) indicate that spore size, basidiomata and tomentum classification were diagnostic features in the definition of three large phylogenetically based groups, called *striatum*, with basidiospores larger than 15 µm and dark-colored basidiome; *ollum*, comprising species with short tomentum grouped in tufts; and *pallidum*, comprising species with long tangled tomentum. However, despite the proposition of using representatives of the seven groups of Brodie, the authors were not able to include taxa from Brodie’s group VI in the ITS analysis, and group IV in the LSU analysis, in addition to using only nine species and one variety in the combined ITS-LSU analysis, less than half proposed initially in the work.

A specific case of discrepancies in the correct position of the species in the phylogeny occurs with *Cyathus setosus*, grouped in the *striatum* group of Zhao et al. (2007) by the ITS region, but not grouping in any other group by the LSU or in the concatenated ITS-LSU analysis. It is thus suggested by the authors that this species may represent a distinct group within the genus, and this exposes the need to expand the sampling, in addition to carrying out new tests.

Silva et al. (2016) suggest the delimitation of a probable new group after the classification of Zhao et al. (2007), named *pedicellatum*, which unites species with a pedicel at the base of the basidiome. Of the four species included in this group, only *Cyathus pedunculatus* and *C. apiculatus* show this structure, while *C. poeppigii* and *C. stercoreus* do not contain this feature, making the group name superficial and non-diagnostic.

The last major study involving molecular biology, not exclusively with *Cyathus*, but with bird’s nest fungi in general, was performed by Kraisitudomsook et al. (2021), who observed that *Nidulariaceae* is monophyletic, clustered with *Squamanitaceae* as potential sister group, and presenting 3 main clades (*Cyathus*, *Nidula-Nidularia* and *Crucibulum*) and two isolated branches of *Mycocalia*. In the same work it was observed that the *Cyathus* clade presents inconsistencies among internal groups, requiring further analysis with type materials and more robust molecular data (Kraisitudomsook et al. 2021), as already observed by Zhao et al. (2007).

Thus, this work aims to expand and understand the internal phylogenetic relationships among the fungi of the genus *Cyathus* and examine how these relationships are reflected in taxonomic characterization, through molecular analyses covering most of the species in the group, based on materials obtained from type specimens deposited in major fungal collections worldwide, and to expand the sampling with tropical species.

## METHODS

### Sampling

The samples used in this study were obtained through loan from the following collections: BIOTEC Bangkok Herbarium & Fungarium (BBH—Thailand), U.S. National Fungus Collections, USDA-ARS (BPI—USA), Herbarium PH, Academy of Natural Sciences of Drexel University (PH—USA), Canadian National Mycological Herbarium (DAOM—Canada), Royal Botanic Gardens (K—United Kingdom), Real Jardín Botánico (MA—Spain), Muséum National d'Histoire Naturelle (PC—France), Herbarium UFRN-Fungos, Departamento de Botânica e Zoologia (UFRN-Fungos—Brazil), and Herbarium of the National Museum of Nature and Science (TNS—Japan). Additional materials collected in Brazil and deposited in the collections of the Lauro Pires Xavier Herbarium (JPB—Universidade Federal da Paraíba), Pe. Camille Torrend Herbarium (URM—Universidade Federal de Pernambuco), and UESC Herbarium (HUESC—Universidade Estadual de Santa Cruz) were also employed in this study. Rare or type species deposited in the collections CAL (India), HKAS, HMAS, MHSU, SWFC (China), AH (Spain), CO (Colombia) and MEXU (Mexico) were not obtained due to the unavailability of those institutions to respond the requests, or the absence of loan programs for rare, old samples or type collections.

DNA extraction from the type specimens was prioritized, but in some cases where it was not possible, the isotype, paratype, or additional material correctly identified and deposited in an international or Brazilian herbarium, preferably collected at or near the type locality, was used. When even under these conditions it was not possible to analyze the materials, samples were requested from the collections according to the following priority: (1) same country as the type, (2) neighboring or nearby countries, or (3) same continent. Materials from continents other than the type specimens were also analyzed to expand the sampling of potentially cosmopolitan species.

### DNA extraction, PCR amplification and sequencing

The initial analyses of samples from European and American herbaria collections were performed in Brazil at the Plant Molecular Genetics Laboratory (Departamento de Biologia Celular e Genética, Universidade Federal do Rio Grande do Norte, Brazil). DNA was extracted using the Whatman® FTA Classic Card (GE Healthcare Life Sciences), following the manufacturer's instructions. Of each sample, one peridiole was selected and submerged in an Eppendorf tube with 20 µl KOH 3% added, for 24 h. PuReTaq Ready-To-Go® PCR Beads (Amersham-Pharmacia Biotech) were used for PCR (Polymerase Chain Reaction) following Martín and Vinka (2000). ExoSAP-IT™ PCR Product Cleanup Reagent (USB Corporation, OH, USA) was used for purification of the amplifications, following the manufacturer’s instructions.

Asian collections and some European and American specimens were analyzed at the Botany Department, National Museum of Nature and Science, Japan, using the following DNA extraction protocol: peridioles stored in DMSO buffer were macerated in liquid nitrogen using a pistil (Hosaka 2009), and transferred to new 1.5 ml tubes. A modified CTAB buffer (Hosaka and Castellano 2008) was added, and the material followed the extraction protocol and glass milk purification method (Hosaka 2009; Hosaka and Castellano 2008). Amplifications were performed with EmeraldAmp MAX PCR Master MIX (TaKaRa BIO Inc.).

Ten different primers were tested for amplification of the DNA regions, four of them designed exclusively for the ITS and LSU regions of *Cyathus*: ITS-CyR3, ITS-CyF4, LSU-CyR and LSU-CyF (Table 2). The need to design new primers, two of them in fact low degenerated primers (ITS-CyR3 and ITS-CyF4, Table 2), occurred because most samples were collected during the nineteenth century, presenting DNA in fragments, making the amplification of the DNA regions a challenge. The use of low degenerate primers in the ITS region allows an increase in the amplification efficiency, since this is a fast-evolving region and presents similar, but not identical, base pair sequences among the species of the genus.

**Table 2:** Primers used for DNA amplification of *Cyathus* species

Locus	Primer name	Sequence (5′ → 3′)	Sense	References
nrITS	ITS1	TCCGTAGGTGAACCTGCGG	F	White et al. (1990)
	ITS1F	CTTGGTCATTTAGAGGAAGTAA	F	Gardes and Bruns (1993)
	ITS2	GCTGCGTTCTTCATCGATGC	R	White et al. (1990)
	ITS4	TCCTCCGCTTATTGATATGC	R	White et al. (1990)
	ITS-CyR3	ACCYAATAGAAGCRGYHCAA	R	This study
	ITS-CyF4	ADTTGAWGTCRGCTYTCKCTG	F	This study
28S (nrLSU)	LR0R	ACCCGCTGAACTTAAGC	F	Vilgalys and Hester (1990)
	LR5	TCCTGAGGGAAACTTCG	R	Vilgalys and Hester (1990)
	LSU-CyR	ATGCCAACATCCGAAGCAC	R	This study
	LSU-CyF	AAGGGAAACGCTGGAAGTCA	F	This study

**Fig. 1:**
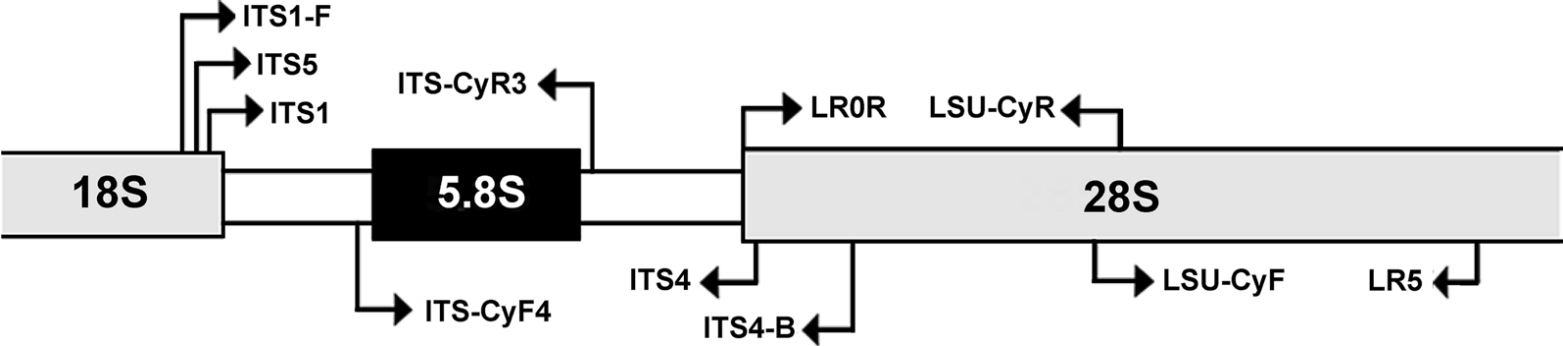
Schematic map of primer position and combinations used for DNA amplification of *Cyathus* rDNA regions**.** The ITS-CyR3 and ITS-CyF4 were designed for the amplification of part of the ITS region of rDNA, while primers LSU-CyR and LSU-CyF were designed for the amplification of part of the LSU region (28S) of rDNA. The four new primers designed in this study were tested only with the genus *Cyathus* and together with primers already known in the literature, as shown in Table 2

The recommended combination for the common primers was used initially as follows: ITS1F (Gardes and Bruns 1993) or ITS1 (White et al. 1990) was used with ITS4 (White et al. 1990) for the Internal Transcribed Spacer of Ribosomal DNA region (ITS1 and IT2, including the 5.8S gene), and LR0R and LR5 (Vilgalys and Hester 1990) for the Large Subunit of Ribosomal DNA (nuc-LSU or 28S). When those combinations did not give good amplifications, newly designed primers were then employed. For the ITS region, ITS-CyR3 primer is located just after the 5.8S region in *Nidulariaceae*, and could be used as the reverse primer in conjunction with ITS1/ITS1F/ITS5, while ITS-CyF4 primer binds just before the 5.8S region and could be used as the forward primer in conjunction with ITS4/ITS4B (Fig. 1). For the LSU region, the LSU-CyR primer binds closely to the central portion of the LSU region (when compared to sequences using the LR0R-LR5 primers) and could be used as reverse primer in combination with LR0R, while the LSU-CyF primer binds just before the area where LSU-CyR binds and could be used as forward primer in conjunction with LR5 (Fig. 1).

PCR reaction conditions were performed following Martín and Vinka (2000). The temperature gradient always consists of an initial 5-min denaturation step at 94 °C and a final extension of 10 min at 72 °C. For the ITS region, PCR was performed in two stages: 5 cycles of 30 s at 94 °C (denaturing stage), 30 s at 54 °C (annealing stage), 1 min at 72 °C (extending stage) and 33 cycles of 30 s at 94 °C (denaturing stage), 30 s at 48 °C (annealing stage), 1 min at 72 °C (extending stage). For the LSU region, PCR was performed with the same two stages and temperature gradient for the ITS, except for the extending stage of the second stage, with 1 min and 30 s at 72 °C. For both regions, final hold consisted of a temperature of 4 °C.

PCR products were purified with ExoSAP-IT® (USB), according to the manufacturer’s instructions, and then sent to be sequenced by Macrogen (Seoul, South Korea) with the same primers used for each amplification. Samples analyzed in Japan were sequenced in the Botany Department of the National Museum of Nature and Science (Tsukuba, Japan), through the BigDye Terminator Cycle Sequencing Kit (Applied Biosystems) with the same primers used for DNA amplification. The online tool “Standard Nucleotide BLAST” (National Center for Biotechnology Information, USA, available at http://blast.ncbi.nlm.nih.gov/Blast.cgi) was used to confirm if the sequences obtained were representatives of the genus *Cyathus*. All the new sequences were deposited in GenBank® (http://www.ncbi.nlm.nih.gov/).

### Phylogenetic analyses

Type sequences published by Zhao et al. (2008) were obtained from NCBI (GenBank) and newly generated sequences in this study were also submitted to GenBank under the accession numbers indicated in Table 3. Three datasets were constructed: a dataset with aligned ITS sequences of 37 species, a dataset with aligned LSU sequences of 36 species, and a combined ITS/LSU dataset, comprising sequences of 28 species. All datasets were aligned using Clustal X algorithm and adjusted manually in MEGA: Molecular Evolutionary Genetics Analysis v. 7.0.14 (Kumar et al. 2016). Inconsistent nucleotide positions were filled with “N” and alignment gaps were marked with “–”, following Accioly et al. (2019). Three other bird’s nest fungi species—*Crucibulum laeve* (Huds.), *Mycocalia aquaphila* R. Cruz, L.T. Carmo, M.P. Martín, Gusmão & Baseia, *Nidula* sp.—and *Cystoderma amianthinum* (Scop.) Fayod were included as an outgroup. Phylogenetic analyses were conducted for all datasets under maximum parsimony (MP) and Bayesian.

**Table 3:** *S*pecimens analyzed in molecular analysis. GenBank accession numbers for each region of the sequences included in this study

Species	Country	ITS	LSU
*Cystoderma amianthinum*	Australia	KP311459	KP311339
*Mycocalia aquaphila*^type^	Brazil	MG836281	MG836282
*Crucibulum laeve*	Japan	KX906249	KX906256
*Nidula* sp.	Japan	KX906248	KX906255
*Cyathus africanus*^type^	Tanzania	DQ463347	DQ463330
*Cyathus albinus*^type^	Brazil	KY176371	KY176372
*Cyathus amazonicus*^type^	Brazil	KY495280	KY495281
*Cyathus annulatus* var. *annulatus*^type^	Canada	DQ463351	DQ463332
*Cyathus aurantiogriseocarpus*^type^	Brazil	KX966026	KX966027
*Cyathus badius*^type^	Japan	KX906250	KX906257
*Cyathus berkeleyanus*	China	DQ463355	–
*Cyathus colensoi*^type^	India	DQ463344	–
*Cyathus crassimurus*^type^	Hawaii	DQ463350	–
*Cyathus discoideus*^type^	China	KY652080	–
*Cyathus earlei*	Brazil	–	KY652083
*Cyathus gracilis*	Japan	KY652081	KY652084
*Cyathus griseocarpus*^type^	India	–	DQ463324
*Cyathus guandishanensis*^type^	China	–	DQ463329
*Cyathus helenae*^type^	Canada	–	DQ463334
*Cyathus hookeri*	China	DQ463346	–
*Cyathus hortensis*^type^	Brazil	KX906252	–
*Cyathus ibericus*^type^	Spain	KX858597 (ITS-LSU)	
*Cyathus isometricus*^type^	Brazil	MF595985	MF595986
*Cyathus jiayuguanensis*^type^	China	DQ463341	DQ463325
*Cyathus lanatus*^type^ (former type species of *C. olla* f. *lanatus*, synonymized by Zhao et al. (2007)	USA	–	DQ463337
*Cyathus lignilantanae*^type^	Cape Verde	KX906254	KX906258
*Cyathus limbatus*	Guyana^type^	KY176373	–
	Brazil	–	KY176374
*Cyathus magnomuralis*^type^	Brazil	KX906251	KX906259
*Cyathus minimus*	Japan	KY652082	KY652085
*Cyathus olla* f. *olla*	Canada	DQ463345	DQ463327
*Cyathus olla* f. *anglicus*^type^	USA	–	DQ463326
*Cyathus olla* f. *brodiensis*	China	DQ463343	–
*Cyathus pallidus*	China	DQ463356	DQ463336
*Cyathus parvocinereus*^type^	Brazil	KX906253	KX906260
*Cyathus poeppigii*	China	–	DQ463339
*Cyathus pygmaeus* (former type species of *C. gansuensis*, synonymized by Zhao et al. (2006)	China	DQ463348	DQ463335
*Cyathus pyristriatus*^type^	Thailand	KU865513	KU865514
*Cyathus renweii*^type^	China	DQ463352	DQ463333
*Cyathus setosus*^type^	Jamaica	DQ463349	DQ463331
*Cyathus stercoreus*	China	DQ463354	DQ463338
	Brazil	KY176376	KY176377
*Cyathus subglobisporus*^type^	Thailand	EF613553	EF613554
*Cyathus triplex*	China	DQ463353	–
*Cyathus uniperidiolus*	India	MN398297	MN398298

Type species sequences are marked with the superscript “type” name. Accession numbers underlined are from newly generated sequences. Absences of sequences are marked with “–”

MP analyses were conducted in software PAUP* ver. 4.0a169 (Swofford 2003), where phylogenetic trees were calculated by heuristic search with the Tree Bisection Reconnection (TBR) algorithm and Multrees option applied by default in PAUP*. Gaps were treated as missing data and trees were obtained by 10 × randomized stepwise addition, with a bootstrap of 10,000 replicates (MPbs) and the number of trees obtained in each replication limited to 1000. The consensus tree included 50% compatible groups by majority-rule consensus, and the homoplasy index (HI) (Archie 1996), retention index (RI) (Farris 1989), consistency index (CI) (Kluge and Farris 1969) and rescaled consistency index (RC) (Farris 1989) were obtained.

Bayesian analysis was performed using MrBayes v.3.2.2 (Ronquist et al. 2012) implemented in CIPRES Science Gateway v3.3 (Cyberinfrastructure for Phylogenetic Research web portal, Miller et al. (2010). The best nucleotide substitution model for each partition (ITS, ITS/LSU and LSU) was selected by the software jModelTest2 (Darriba et al. 2012) according to the AIC (Akaike Information Criterion) and AICc (Corrected Akaike Information Criterion). Twenty million generations were performed, with trees sampled every 1000 generations. The Average Standard Deviation of Split Frequencies (AvgStdDev) was used to discard a portion of the trees at the burn-in stage when the value was above 0.01. The convergence between the runs was indicated when the potential scale reduction factor (PSRF) approached 1.0 (Gelman and Rubin 1992). The minimum and mean values for estimated sample size (ESS) were also analyzed. ESS values below 100 may indicate that the convergence parameters were under-sampled. When the node shows a BPP ≥ 0.95 and/or BS ≥ 80%, it was considered significantly supported.

The obtained trees were visualized in software FigTree v. 1.4.2 (http://tree.bio.ed.ac.uk/software/figtree/), exported as PDF and edited in Corel Draw® Graphics Suite 2021 software.

## RESULTS AND DISCUSSION

For this study, 39 species of *Cyathus* from 41 samples were used, with 26 of them belonging to a nomenclatural type category (e.g. holotype, isotype, epitype, ex-type, or others). Sequences for 12 type specimens had already been done by Zhao et al. (2007, 2008). Additional sequences from types published by other research groups in recent years, such as *Cyathus ibericus* (Crous et al. 2016), *C. pyristriatus* (Hyde et al. 2016) and *C. uniperidiolus* (Boonmee et al. 2021), and sequences from types published by this group, such as *C. aurantiogriseocarpus* (Crous et al. 2017a, b), *C. isometricus* (Crous et al. 2017a, b), *C. badius*, *C. hortensis*, *C. magnomuralis*, *C. parvocinereus* (Cruz et al. 2018), *C. albinus*, *C. amazonicus*, *C. lignilantanae* and *C. limbatus* (Accioly et al. 2018) supplement the dataset used in this review. In addition, this study generated sequences for the type of *C. discoideus*, and sequences from other species like *C. earlei*, *C. gracilis* and *C. minimus*.

The ITS dataset includes 37 taxa, comprising 33 *Cyathus* species, and the LSU dataset consists of 36 taxa, 32 of them *Cyathus* specimens. For the concatenated dataset, 28 taxa were included, 24 of them *Cyathus* species. *Crucibulum laeve*, *Cystoderma amianthinum*, *Nidula* sp. and *Mycocalia aquaphila* were used as outgroups in all analyses.

Maximum Parsimony analyses for the ITS region returned 547 characters out of a total of 950 characters, 198 of them parsimoniously informative and 205 parsimoniously uninformative. The parsimony analyses resulted in a most parsimonious tree with 721 steps (CI = 0.6962, RI = 0.7296, RC = 0.5080, HI = 0.3037). For the LSU dataset, Maximum Parsimony analyses returned 838 characters, 75 of them parsimoniously informative and 84 parsimoniously uninformative. The parsimony analyses returned the most parsimonious tree for the LSU dataset with 226 steps (CI = 0.7122, RI = 0.7022, RC = 0.4773, HI = 0.3588). For the ITS-LSU dataset, Maximum Parsimony analyses returned 1688 characters, 272 of them parsimoniously informative and 248 parsimoniously uninformative. The parsimony analyses returned the most parsimonious tree for the concatenated dataset with 796 steps (CI = 0.7963, RI = 0.7974, RC = 0.6112, HI = 0.2334).

Bayesian analyses for the ITS, LSU and concatenated datasets returned a similar topology. We ran 3 Bayesian analyses under different evolutionary models, chosen through jModelTest 2 (Miller et al. 2010): TPM2uf + G for ITS, GTR + G for LSU and GRT + I + G for the concatenated dataset. Bayesian analyses gave a best tree with -lnL = -4.518.803 for ITS dataset, -lnL = -2.473.334 for LSU dataset and -lnL = -6.577.357 for the concatenated dataset.

Since Zhao et al. (2007) were unable to include taxa from the group VI—*poeppigii* in the ITS dataset, and IV—*gracilis* in the LSU dataset, based on Brodie’s classification (Brodie 1975), comparative analyses between infrageneric groups in both classifications were not completely solved. With the sequences used in this work, this lack was solved for group VI in the ITS dataset, with the addition of the type sequence of *Cyathus limbatus*, and for group IV in the LSU, with the sequence of *C. gracilis*. Although representatives of all seven groups were included in the separate analyses by gene region, the absence of a concatenated ITS-LSU sequence for *Cyathus limbatus* or any other species from Brodie's group VI—*poeppigii* (Brodie 1975) continued to make it impossible to include representatives of this group in the concatenated analysis.

Sequences from GenBank from specimens with doubtful identification (specimens identified only as "sp.", specimens obtained exclusively from culture media without clear morphological identification, and specimens considered common and cosmopolitan that were generically described) were avoided to avoid compromising the support of the trees and the positioning of clades. Uncertain sequences for these “common species”, such as *Cyathus striatus*, are potentially compromising for phylogeny, since in herbaria it is frequent to find specimens with this identification made only because they present striations in the basidiome. The inclusion of these specimens could cause the name *C. striatus* to be present in multiple clades, as occurred in the ITS tree for *C. ibericus* in Crous et al. (2016).

Taking a more conservative view, it is possible to infer that the sequences of *Cyathus striatus* present this divergence in the clades because it is a possible cryptic species, but during the morphological review carried out in this work, some samples of *C. poeppigii* and *C. montagnei* were identified as *C. striatus* in different herbaria only because of their plications in the peridium. We cannot deny the possible crypticity of *C. striatus*, but the existence of a species complex in *C. striatus* can only be proven if the specimens are correctly identified and with the morphological features similar to the type specimen, taking as a parameter also the slight differences between the European and American materials, as cited by Lloyd (1906) and Brodie (1975).

A divergent case is observed in the specimens of *Cyathus stercoreus* included in this review. Samples from Asia and South America cluster with higher support in Maximum Parsimony and Bayesian analyses, even collected from geographically distant areas. This clustering was also observed with the same specimen from Asia and other inserted sequences from other localities in Crous et al. (2016), also with high support in the clades.

In addition, species sequences of species synonymized or corrected by Zhao et al. (2007) were used with correct identification: *Cyathus africanus* var. *latisporus* synonymized as *C. jiayuguanensis*; the Chinese material of *C. pygmaeu*s that was previously referred to as the holotype of *C. gansuensis*; and *C. olla* f. *lanatus,* which is now described as *C. lanatus* (Zhao et al. 2007).

**Fig. 2:**
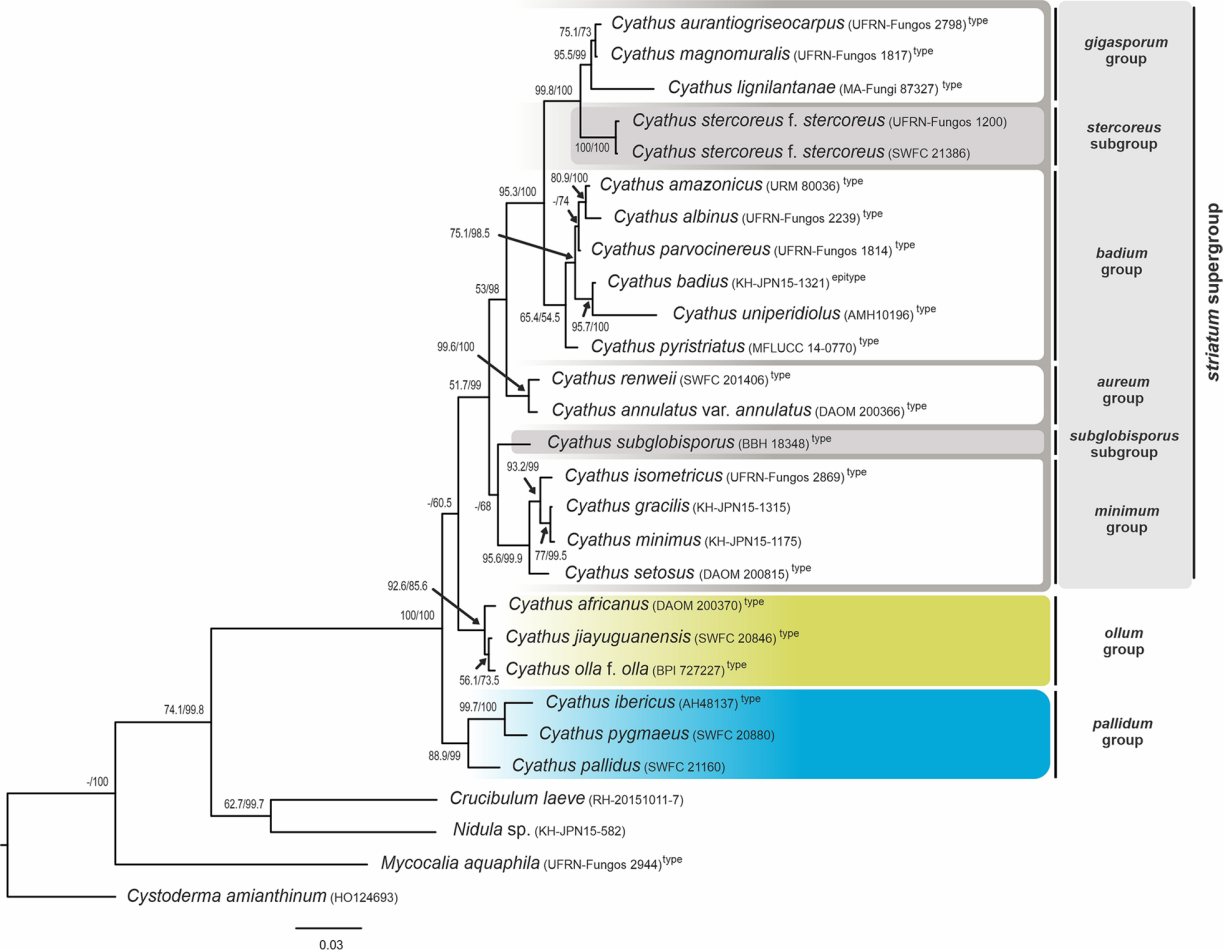
Phylogenetic tree of *Cyathus* obtained by Bayesian analysis and inferred through concatenated sequences of the ITS-LSU regions of rDNA. The numbers in the branches refer to Maximum Parsimony Bootstrap (BS) support, and Bayesian posterior probability (pp). Type species sequences are marked with the superscript “type” name. Values less than 50% in BS or pp are referenced with “–”. Scale bar indicates the number of nucleotide substitutions per site

**Fig. 3:**
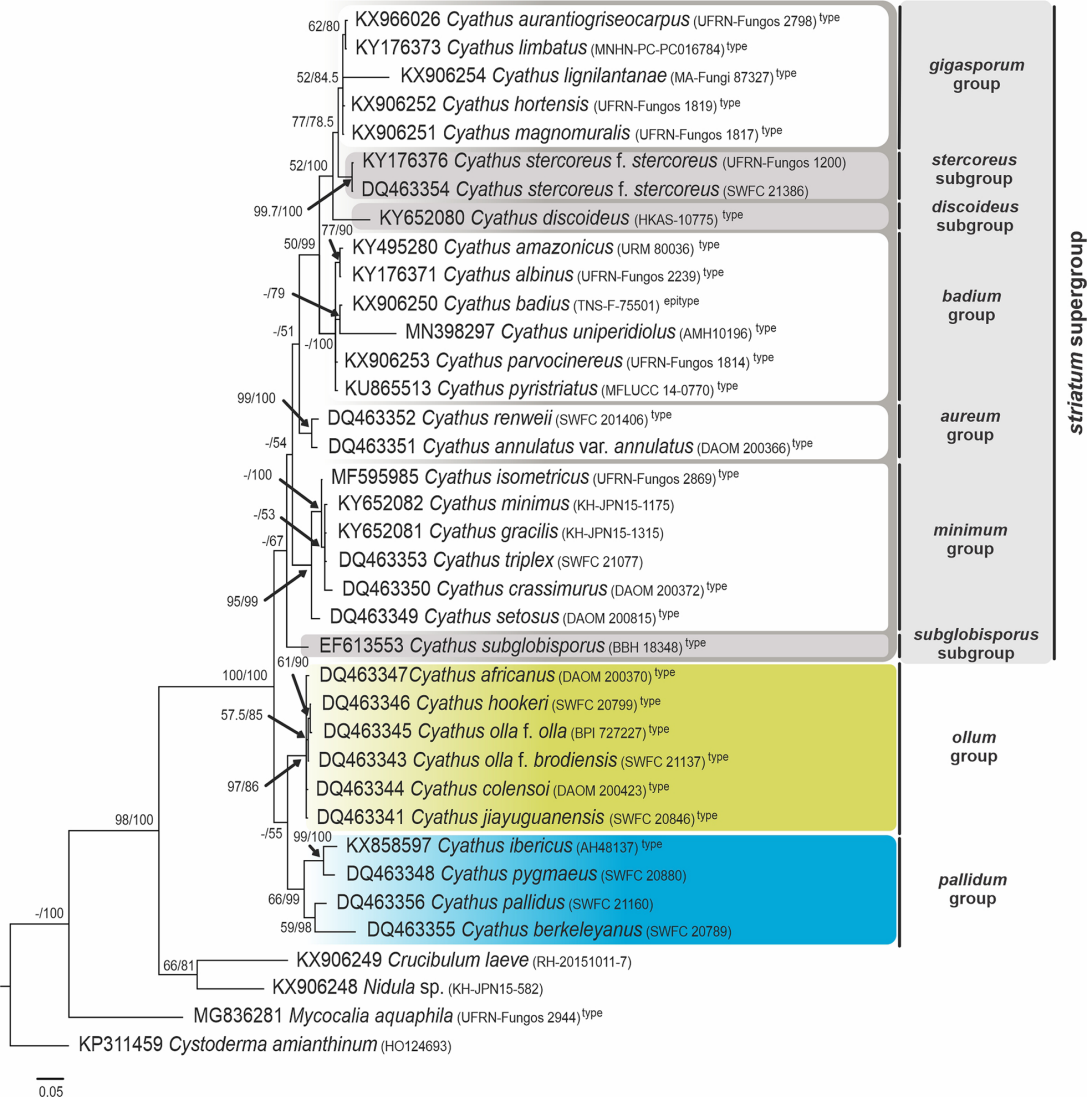
Phylogenetic tree of *Cyathus* obtained by Bayesian analysis and inferred through concatenated sequences of the ITS region of rDNA. The numbers in the branches refer to Maximum Parsimony Bootstrap (BS) support, and Bayesian posterior probability (pp). Type species sequences are marked with the superscript “type” name. Values less than 50% in BS or pp are referenced with “–”. Scale bar indicates the number of nucleotide substitutions per site

**Fig. 4:**
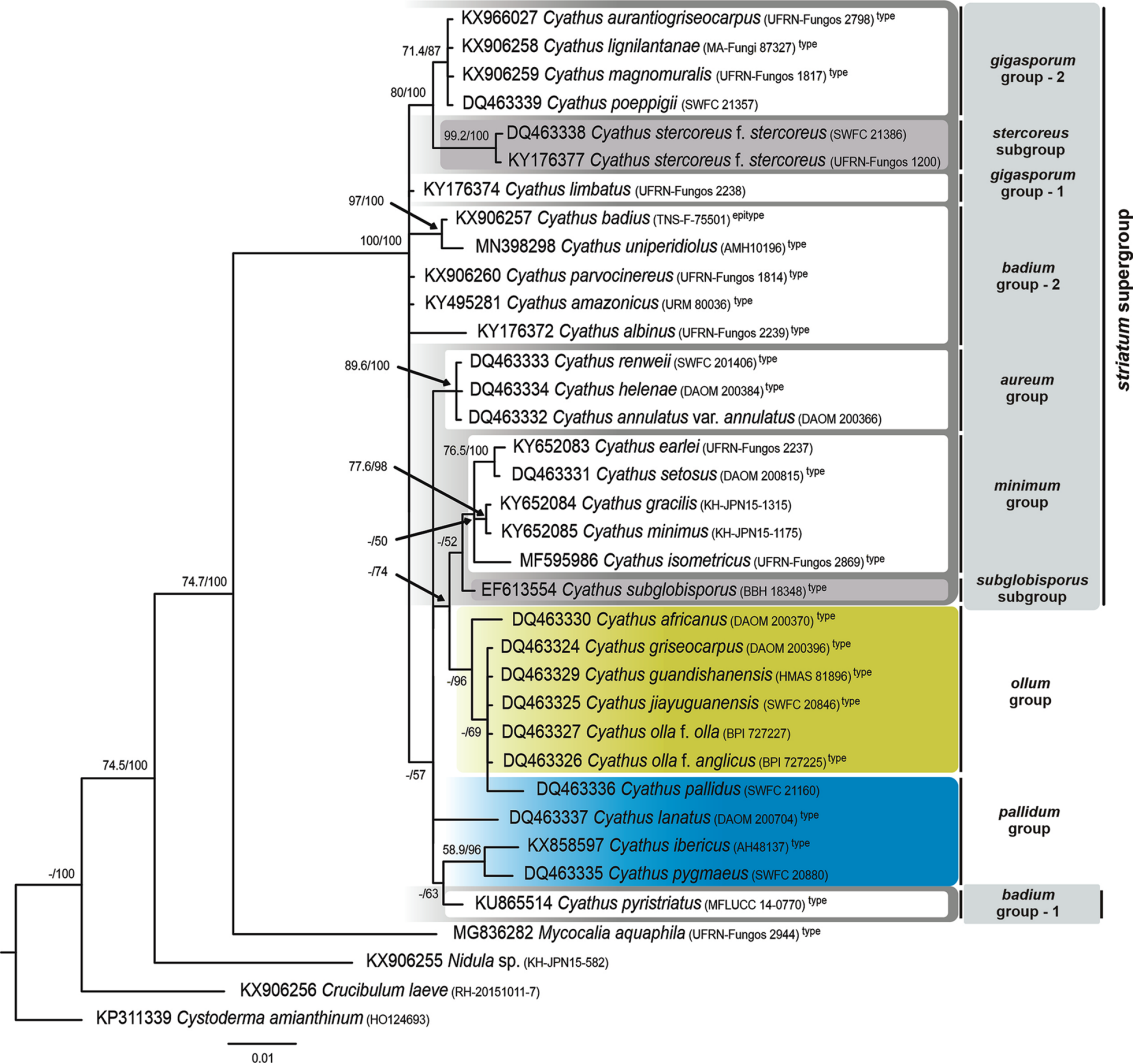
Phylogenetic tree of *Cyathus* obtained by Bayesian analysis and inferred through concatenated sequences of the LSU region of rDNA. The numbers in the branches refer to Maximum Parsimony Bootstrap (BS) support, and Bayesian posterior probability (pp). Type species sequences are marked with the superscript “type” name. Values less than 50% in BS or pp are referenced with “–”. Scale bar indicates the number of nucleotide substitutions per site

Regarding the tree topologies, the insertion of new sequences elucidated some of the internal clade organization when compared to the work of Zhao et al. (2007), but the distribution of the majority groups known since 2007 was maintained. The phylogenetic trees resulting from ITS, LSU and concatenated ITS-LSU datasets can be seen in Figs. 2, 3 and 4.

The ITS tree and the tree for ITS-LSU data showed clusters with better support and similar topology. In contrast, the LSU analysis exhibited large polytomy among many of the groups, together with complex clusters within *Cyathus*, which was expected since LSU sequences are more conserved among species of the same genus.

### Molecular phylogeny

#### Pallidum group

One of the groups was previously defined by Zhao et al. (2007) as having basidiospores smaller than 15 µm, basidiome light yellow, orange, golden, gray or yellowish brown, base of the peridium not constricted and thick tomentum. With the expanded morphological types analysis, this group is defined as “basidiospores smaller than 15 µm and without apiculus; exoperidium and endoperidium unmarked (smooth), sometimes inconspicuous internally; peridiole without tunic, with single-layered cortex”.

The *pallidum* group comprises the species *Cyathus berkeleyanus* (only in ITS), *C. lanatus* (former *C. olla* f. *lanatus*—only in LSU), *C. pygmaeus* (Chinese sample, former type species of *C. gansuensis*), *C. ibericus* and *C. pallidus*. The clade *Cyathus pygmaeus*—*C. ibericus* is supported in all proposed analyses (ITS: 99 BS/100% pp; LSU: 58.9BS/96% pp; ITS-LSU: 99.7 BS/100% pp). *Cyathus pallidus* and *C. berkeleyanus*, initially described as two forms of the species *C. microsporus* by Tulasne and Tulasne (1844), are now considered morphologically distinct species, and their sequences also reproduce this separation in the ITS analysis (ITS: 59 BS/98% pp; no LSU sequences of *C. berkeleyanus* was obtained). The positioning of *Cyathus lanatus* was not supported exactly inside the *pallidum* group in the LSU tree, but in a polytomy. However, based on the morphology of the species, the low definition of the *pallidum* group in this genetic region, and the position in the LSU tree in Zhao et al. (2007), we considered this sample as part of the *pallidum* group (< 50BS and < 50%pp).

As regards the positioning of the group within *Cyathus*, the *pallidum* group forms a clade with the *ollum* group in the ITS analysis (< 50 BS/55% pp), and the most basal clade of the genus in the ITS-LSU concatenated analysis (88.9 BS/99% p.p.). Finally, the real doubts about the correct positioning of type species within this group are *C. pallidus* and *C. berkeleyanus*, two Chinese samples identified by Zhao et al. (2007), and even with the species having been indicated as “morphologically confirmed” by the authors, the type localities of both species are geographically distant (Cuba and Brazil, respectively) and can be a focus for future studies.

#### *Ollum* group

The second group of the previous classification proposed by Zhao et al. (2007) was defined as having basidiospores smaller than 15 µm, and basidiomata with yellowish colors (yellow, gold, gray or yellowish brown), like the *pallidum* group, in addition to the exclusively constricted peridium base, and provided with short and soft tomentum. Based on the expanded morphological types analysis, the group can be defined as: “basidiospores smaller than 15 µm, with rare exceptions reaching up to 18 µm, sometimes with an apiculus and commonly presenting an ovoid shape; exoperidium and endoperidium commonly unmarked (smooth), sometimes inconspicuous internally; emplacement cottonous; single-layered cortex with yellowish or bronze tunic”.

The *ollum* group did not change in its structure and species composition after the classification by Zhao et al. (2007), with no new taxa included in the group. The group is formed by *Cyathus hookeri*, *C. olla* f. *brodiensis*, *C. colensoi* (both exclusive to ITS), *C. griseocarpus*, *C. guandishanensis*, *C. olla* f. *anglicus* (both exclusive to LSU), *Cyathus africanus*, *C. jiayuguanensis* and *C. olla* f. *olla*. As a clade, *ollum* is strongly supported in the ITS analyses (97 BS/86% pp), probably as a sister-group with *pallidum* (< 50 BS/55% pp), and in the ITS-LSU concatenated analysis (92.6 BS/85.6% pp) appears as basal to the entire former *striatum* group (< 50 BS/60.5% pp) proposed by Zhao et al. (2007). In the LSU analysis, the *ollum* group has inconsistencies in its composition, including the presence of *C. pallidus* (*pallidum* group) inserted in a huge polytomy with other species, but it is a result of the low definition of clades in this genetic region.

The species inside the *ollum* group are phylogenetically difficult to delimit, but all of them are morphologically distinct. Despite these morphological differences, the species of the *ollum* group differ from each other by only 1 to 3 bp; among the 802 bases present in the ITS alignment, there is 3 to 6 bp of difference between the 802 bp in the LSU alignment, and 1 to 8 bp difference between the 1606 bp in the ITS-LSU concatenated alignment, indicating that the group probably needs to be better understood using other genetic regions.

#### *Striatum* supergroup (former *striatum* group)

The broadest group in the classification by Zhao et al. (2007) was originally delimited by species with basidiospores larger than 15 µm and fructifications with brown, reddish brown or dark brown color. This review proposes that this large group be treated as a supergroup, segregated into four other groups and tree subgroups, and defined exclusively by the basidiospores larger than 15 µm.

#### *Minimum* group

“Basidiospores between 15 and 20 µm, elliptical and without apiculus; peridium with short tomentum, from 0.2–0.8 mm long, woolly to hirsute; exoperidium yellowish brown (honey yellow, clay brown) to reddish brown; thin mouth (< 0.5 mm in height) in a continuous pattern; internal and external wall smooth to inconspicuously plicated; peridioles brownish, double-layered cortex and with exocortex bronze or brownish, rarely black”. The group name was chosen based on the minute measurements of the tomentum and mouth, in addition to the species *Cyathus minimus*, which most represents the characteristics.

The group is composed of the type species of *Cyathus crassimurus*, *C. isometricus* and *C. setosus*, as well as samples of *C. earlei* (Brazil), *C. gracilis* and *C. minimus* (Japan) and *C. triplex* (China). The entire grouping presents good support in the analyses of ITS (95 BS/99% pp), and ITS-LSU (95.6 BS/99.9% pp), but it presents low support in the LSU (< 50 BS/50% pp). The internal group *C. gracilis*—*C. minimus* is presented only in LSU and ITS-LSU (LSU: 77.6 BS/98% pp; ITS-LSU: 77 BS/99.5% pp) and forms a polytomy with *C. triplex* and *C. crassimurus* in the ITS (< 50 BS/53% pp). *Cyathus earlei* and *C. setosus* are grouped in the LSU tree with good support (76.5 BS/100% pp).

Important conclusions include the fact that previous analyses of specimens of *C. earlei* from Brazil indicate that Brazilian samples have an identical morphology with the type species from Puerto Rico, except for the peridiole cortex: double-layered, well-delimited, with compact white intermediate hyphae in the Brazilian material, while is subhomogeneous in the type material. This single feature seems to be informative inside the genus, supporting the idea that the Brazilian specimens could be a new variety within the species in future molecular analyses. Another species to mention is *Cyathus triplex*: the position of this species in the tree can also be readjusted in future analysis, since the type is from the Americas and this material, sequenced by Zhao et al. (2007), comes from Asia and could not be analyzed morphologically by us. Another two species, *Cyathus gracilis* and *C. minimus*, were included in the analysis because they are from localities near to the type, and unlike the small morphological variations observed in *C. earlei*, and geographical differences observed in *C. triplex*, the specimens of *C. gracilis* and *C. minimus* are equally compatible with their respective nomenclatural types (Philippines and China, respectively).

#### *Subglobisporus* subgroup

Basal to the entire *striatum* supergroup in the ITS tree (< 50 BS/67% pp), the clade composed exclusively by *Cyathus subglobisporus* forms a sister group that has low support with the *minimum* group (LSU: < 50 BS/52% pp; ITS-LSU: < 50 BS/68% pp). In preliminary analyses, *Cyathus subglobisporus* was grouped within the clade now proposed as the *minimum* group; however, when new sequences were included, this species was separated from the others by: “basidiospores globose to slightly elliptical; peridium strongly hirsute with rigid and regular tufts of tomentum; exoperidium mustard brown provided with orange tomentum; endoperidium inconspicuously to conspicuously plicated; and bronze peridioles”.

#### *Aureum* group

“Basidiospores 15–20 µm, sometimes reaching 30 µm, elliptical to cylindrical, without apiculus; peridium hirsute with a yellowish-brown tomentum on a brownish mesoperidium; peridioles with bronze gleba, single-layered cortex and brownish tunic”. The group name was chosen based on the bronze/golden colors of the tomentum, bronze gleba and brownish tunic present in both species.

The *aureum* group is composed of the type species of *C. annulatus* var. *annulatus*, *C. helenae* and *C. renweii*, and their positioning has been known since the classification by Zhao et al. (2007). In our study, *annulatum* is strongly supported in all analyses (ITS: 99 BS/100% pp; LSU: 89.6 BS/100% pp; ITS-LSU: 99.6 BS/100% pp). The outstanding difference between the dark-colored peridium and the lighter-colored tomentum, together with the bronze color gleba and brownish tunic, gives this group its unique features. Of the three species, only the type of *C. renweii* was not re-analyzed morphologically, and it is the only species that presents spores that reach > 20–30 µm in length, values observed commonly in the *gigasporum* group. Until reanalysis, we will assume that the spore values ​​for *C. renweii* are probably wrong or are an exclusive feature for this species.

#### *Badium* group

“Basidiospores 12–20 µm, sometimes reaching 30 µm, globose to elongated, rarely ovoid in some species; peridium hirsute, brownish; peridioles grayish-brown to reddish-brown, 2–3 mm in diameter, with double-layered cortex, varying from subhomogeneous with grayish-white intermediate hyphae, to non-subhomogeneous double-layered with darkened intermediate hyphae”. The group name was chosen based on the brown to reddish-brown pattern in the sample, in addition to the species *Cyathus badius*, which most represents the characteristics.

The *badium* group is composed of the species *Cyathus albinus*, *C. amazonicus*, *C. badius*, *C. parvocinereus*, *C. pyristriatus* and *C. uniperidiolus*. Within the LSU analysis this clade has low support, with *C. albinus*, *C.amazonicus* and *C. parvocinereus* in a polytomy, and *C. pyristriatus* grouping with the clade *C. ibericus*—*C. gansuensis* of the *pallidum* group (< 50 BS/63% pp). In both ITS and concatenate tree, the *badium* group is well supported in the Bayesian Analysis and forms a sister group with most derived groups within *Cyathus*: *gigasporum* group, *discoideus* subgroup and *stercoreus* subgroup (ITS: 50 BS/99% pp; ITS-LSU: 95.3 BS/100% pp). In the most well-defined tree (ITS-LSU), the three Brazilian type species are grouped together: *Cyathus amazonicus* and *C. albinus* from the Amazon forest (80.9 BS/100% pp), together with *C. parvocinereus* from the “Brejos de altitude” (humid enclaves) in the northeastern dry forests, forming a sister group (< 50 BS; 74% pp). *Cyathus amazonicus* and *C. albinus* are also grouped in the ITS tree (77 BS/90% pp).

Another species grouped is *Cyathus badius*—*C. uniperidiolus,* well supported in both analyses (ITS: < 50 BS/79% pp; LSU: 97 BS/100% pp; ITS-LSU: 95.7 BS/100% pp). *Cyathus uniperidiolus* is the most recent *Cyathus* species described for the genus, but the description published by Boonmee et al. (2021) presents many discrepancies with the photos, mainly the peridium form, described as globose to subglobose, but the samples present clearly infundibuliform basidiomata. Looking closely at the figures in that work, is almost certain that what the authors consider as a “globose basidiocarp” is a double-layered cortex with a fissured exocortex, and the strange shape of the peridioles can be a aberrant product of the culture technique used to cultivate this sample. Until we have been able to carry out the analysis of this type material, we suggest that the morphological similarities, such as colors, infundibuliform peridium (clearly seen in “Fig. 196.a” published by Boonmee et al. (2021) and size of the spores indicate that *C. uniperidiolus* must be *C. badius* or a closely related species).

#### *Discoideus* subgroup

Similarly to *Cyathus subglobisporus*, *C. discoideus* was positioned isolated in the ITS analysis, basal to the *stercoreus* subgroup and *gipasporum* group. The subgroup can be defined as: “basidiospores 15–20 µm in length, elliptical; peridioles grayish-brown to sepia, with strongly rugulose surface; exoperidium smooth to inconspicuously plicated, and mouth finely fimbriate in a continuous pattern, becoming intermittent in older basidiomata”. No LSU sequence was obtained from this type material, but morphological characteristics of the type of *C. limbatus* are compatible with characters of the *discoideus* subgroup, and this distribution may change after the addition of new sequences of different type species.

#### *Stercoreus* subgroup

Another isolated species, *Cyathus stercoreus* f. *stercoreus,* is the first species in the tree with huge spores. The subgroup is defined as: “basidiospores > 20 µm, subglobose to slightly elliptical; smooth peridium; peridioles slightly rugulose, with double-layered cortex and no tunic”. Unlike the other groups of the *striatum* supergroup, this species does not present plication in the peridium. A pertinent fact is that even though we used two sequences of the same taxon from distant geographic regions, samples from China and Brazil are grouped together with high support (ITS: 99.7 BS/100% pp; ITS-LSU: 100 BS/100% pp; LSU: 99.2 BS/100% pp). The “splash-cup” dispersion method used by *Cyathus* ejects the peridioles closer than 2 m away from the basidiome, which reduces the potential dispersion area of this fungus. However, as it is a coprophilous species that needs to pass through the gastrointestinal tract of herbivores to complete its life cycle, it is not uncommon for peridioles to be “released” in more distant areas due to bovine transport facilitated by humans, a fact that can be observed in literature records of *Cyathus stercoreus* f. *stercoreus*, since this species is usually associated with pasture or livestock areas. Thus, with the expansion of cattle breeding, the distribution of this species may have quickly followed the dispersal area of cattle, which would explain why specimens located in such distant and distinct environments were grouped together with high phylogenetic support in the analyses performed.

#### *Gigasporum* group

“Basidiospores > 20 µm, sometimes reaching > 30–40 µm, slightly elliptical to elongated; peridium with conspicuous plication; peridiole with smooth surface, double-layered cortex and no tunic”. The group name was chosen based on the huge spore size of the species.

The *gigasporum* group is composed of the type species of *Cyathus aurantiogriseocarpus*, *C. hortensis*, *C. lignilantanae*, *C. limbatus* and *C. magnomuralis,* as well as an exsiccate of *C. poeppigii* described in China by Zhao et al. (2007). The species identified as *C. limbatus* in the LSU tree is a sample from Brazil, distinct from the type species from Guyana in the ITS tree, and for this reason these sequences were not concatenated. Despite being morphologically identical and from near localities, the type species clustered with *C. aurantiogriseocarpus* in the ITS tree (62 BS/80% pp), while the Brazilian sample of *C. limbatus* was inserted in the initial polytomy of *Cyathus* in the LSU tree, out of the *gigasporum* group, which makes more sense considering the spore size. Another hypothesis is that the Brazilian samples are a cryptic species similar to *C. limbatus*, and the type species underwent a process of reversion in the characteristic “spore size”.

Concerning the other species, the morphology does not conflict with the general characteristics of the group. Supports are higher in the concatenated analysis, with *Cyathus aurantiogriseocarpus* and *C. magnomuralis* in a clade with 75.1 BS and 73% pp, forming a sister group with *C. lignilantanae* (95.5 BS/99% pp); however, polytomies occurred in ITS (except for *C. aurantiogriseocarpus—C. limbatus*) and LSU tree. The *gigasporum* group clusters with *stercoreus* subgroup with high support (ITS: 77 BS/78.5% pp; LSU: 80 BS/100% pp; ITS-LSU: 99.8 BS/100% pp).

Silva et al. (2016) suggested the proposition of the “*pedicellatum* group” in a clade that is apparently formed by the *gigasporum* group and *stercoreus* subgroup proposed here, characterizing this group as presenting species with a “constricted base forming or not forming a pedicel”, and having spores “larger than 15 µm, generally larger than 25 µm”. From the species used by Silva et al. (2016), the descriptions indicate that only *C. apiculatus* and *C. pedunculatus* presented the constricted base at the peridium, and when the sequences of these species were included in our dataset, the alignment presented a huge number of insertions, which include numerous gaps in the 5.8S region of the ITS. This is a region extremely conserved not only within *Cyathus* but conserved in *Nidula* sp., *Crucibulum laeve*, *Mycocalia aquaphila* and even in the species *Cystoderma amianthinum* used as outgroup, forcing us to remove this data and wait for a reanalysis of these types. About the insertion of *C. stercoreus* in the “*pedicellatum* group” by Silva et al. (2016), the presence of a pedicel was never found in the literature or in the samples analyzed by us. To conclude this discussion, none of the figures in Silva et al. (2016) makes the presence of a pedicel clear in the basidiomata of all the species of the “group”. Due to all these details, the pedicel-based grouping proposed by Silva et al. (2016) cannot be considered valid.

#### *Identification key to the infrageneric groups of* Cyathus

**Fig. 5:**
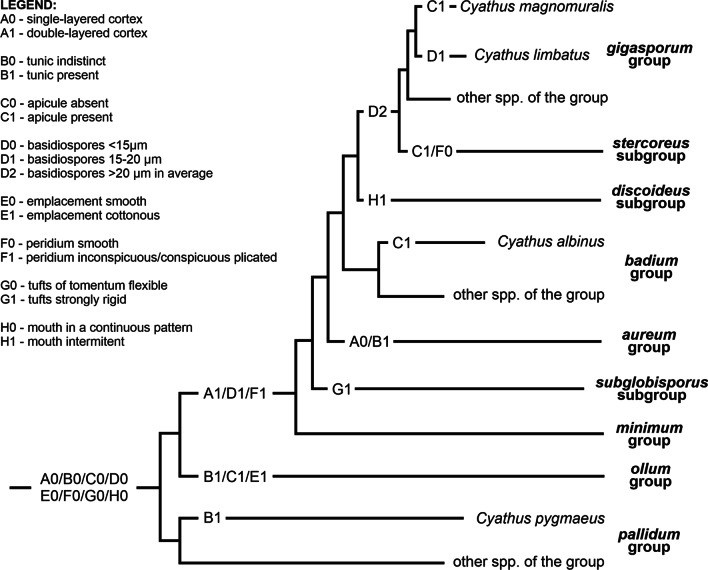
Distribution of the main morphological features of *Cyathus* among molecularly supported groups. The figure distribution follows the ITS-LSU tree, with insertion of the *discoideus* subgroup at the base of the *gigasporum* group—*stercoreus* subgroup clade, and the species *Cyathus limbatus* inside the *gigasporum* group, both according to the ITS tree. The size of the branches does not represent genetic distance

Based on the positioning of infrageneric groups in the phylogeny, it is possible to define which morphological characters are informative for the separation of clades within *Cyathus*. Unlike that indicated by Zhao et al. (2007), some of the characters proposed by Brodie (1975, 1984) can still be used to separate groups, such as the peridiole cortex, peridium plication and spore size, but always in conjunction with other morphological information. Figure 5 demonstrates how the main morphological characters of the group are distributed within the phylogeny. An identification key for groups and subgroups is shown below:

- 1 Single-layered cortex …………………… 2
- Double-layered cortex …………………… 3
- 2 (1) Basidiospores smaller than 15 µm … 4
- Basidiospores 15—20 µm, sometimes reaching 30 µm ………………………………………………. ***aureum*** group
- 3 (1) Tomentum strongly hirsute, rigid/inflexible …………….……………… ***subglobisporus*** subgroup
- Tomentum malleable (woolly or hirsute), not rigid ………………………………………………………. 5
- 4 (2) Emplacement smooth; Peridioles without tunic ……….………………………..…… ***pallidum*** group
- Emplacement cottonous; Peridioles with yellowish or bronze tunic ……………………….... ***ollum*** group
- 5 (3) Mouth in a continuous pattern even in old samples; Peridioles with a smooth to slightly rugulose surface …………………… 6
- Mouth with intermittent pattern in old samples; Peridioles strongly rugulose … ***discoideus*** subgroup
- 6 (5) Basidiospores commonly less than 20 µm ……………………………………….………………….. 7
- Basidiospores bigger than 20 µm ………… 8
- 7 (6) Exocortex and endocortex of the peridiole with different colors; Basidiospores elliptical ……………………………………… ***minimum*** group
- Exocortex and endocortex of the peridiole with the same color; Basidiospores globose to elongated ……………………… ***badium*** group

- 8 (6) Basidiome without plication …..……………………………………….…………. ***stercoreus*** subgroup
- Basidiome conspicuously plicated ……….……………….…………………………….. ***gigasporum*** group

Based on the distribution of morphological characters through the genus *Cyathus*, it is possible to infer that:

1. The “double-layered cortex” appears in the *minimum* group, undergoing an evolutionary reversion exclusively in the *aureum* group;
2. The plesiomorphy “tunic indistinct” appears recurrent in most groups, but in *ollum* and *aureum* groups, in addition to the species *Cyathus pygmaeus* of the *pallidum* group, the apomorphy “tunic present” is observed;
3. The presence of an apiculus is a derived feature that appears in the *ollum* group and *stercoreus* subgroup, in addition to the species *Cyathus albinus* (*badium* group) and *Cyathus magnomuralis* (*gigasporum* group);
4. Spore size increases as clades are more derived, with measurements above 15 µm from the *minimum* group, and measurements above 20 µm between the *stercoreus* subgroup and *gigasporum* group. However, a reversion appears to occur with the species *Cyathus limbatus* within the *gigasporum* group;
5. The “cottonous emplacement” is an autapomorphy of the *ollum* group;
6. Peridium smooth/without plication seems to be the basal characteristic of *Cyathus*, as can be seen in the other genera of Nidulariaceae that have a cup-shaped basidiome: *Crucibulum* and *Nidula*. The presence of inconspicuous or conspicuous plication is a feature that appears in the separation of the *striatum* supergroup from the groups *pallidum* and *ollum*, reversed only in the *stercoreus* subgroup;
7. Only in the *subglobisporus* subgroup the tufts observed on the tomentum are strongly hirsute and extremely rigid. We do not know if it was a protective covering or something similar;
8. Only in the *discoideus* subgroup the hyphae that form the fimbriae of the mouth become intermittent during the maturity of the basidiomata.

Except for the cottonous emplacement, strongly rigid tufts and an intermittent pattern on the mouth fimbriae, all other characteristics were used by Brodie (1975, 1984), but the species groups used by this author are not reproduced in the phylogenetic analyses. In contrast, the grouping proposed by Zhao et al. (2007) was confirmed as the first step towards the new clusters that are delineated after the insertion of new sequences into the alignment.

There is potential to use other gene regions and improve the definition of the results inside the groups that present a large number of polytomies, for example, the *Ollum* group. During the development of this work, tests with other important gene regions for *Gasteromycetes* studies, such as mitochondrial ATP synthase 6 (MT-ATP6), RNA polymerase II (RPB2) and the Translation elongation factor 1-alpha (TEF 1-alpha), were carried out but without success in amplifications and sequencing. This failure may be due to the age of herborization of the samples, with some of the type specimens being more than 70 years old, because the DNA is fragmented, or even because of the need for new adjustments in the protocols used. However, attempts with newly collected samples, and correctly identified as representatives of the type species, may elucidate these phylogenetic data that today are confused in these groups.

Future works primarily using sequences of *Cyathus* type species, or species correctly identified according to the descriptions of the types, in addition to analyses involving the molecular clock and biochemistry, are the way to expand knowledge about the phylogeny, distribution and importance of this genus.

## CONCLUSIONS

Although in recent years the molecular classifications of *Cyathus* have seemed inconsistent with the most updated morphological data used in the identification of species, the inclusion of new sequences proved that the patterns of the tomentum tufts, fimbriae of the mouth and the type of cortex in the peridiole are still good infrageneric morphological delimiters. The initial groups proposed by Zhao et al. (2007), based primarily on color and spore size are still consistent with the phylogeny. With the addition of new sequences, it was possible to further improve the classification of groups within *Cyathus*, which also allowed us to delimit with more certainty which morphological characters can be used to separate the *striatum* supergroup (former *striatum* group in Zhao et al.’s classification (2007)). Further studies are needed to solve the subgroups that are currently composed of "isolated" species. These results shed light on the infrageneric classification of *Cyathus*, proving the importance of revision works being primarily based on type materials or samples authentically identified by experts in the genus.

## Data Availability

The sequences generated and analysed during the current study are available in the Genbank repository (https://www.ncbi.nlm.nih.gov/genbank/). The Dataset generated and analysed during the current study are not publicly available due to other studies being developed by the research group using part of these data, but are available from the corresponding author on reasonable request.
